# Bestandsaufnahme und Evaluierung von Datenquellen im Kontext der psychischen Gesundheit von Kindern und Jugendlichen mit Migrationsgeschichte in Deutschland: Ergebnisse des Projekts STRONGDATA-Kids

**DOI:** 10.1007/s00103-026-04246-2

**Published:** 2026-05-18

**Authors:** Marleen Bug, Carmen Koschollek, Bianka Detering, Veronika Wiemker, Julia Weiss, Kayvan Bozorgmehr, Claudia Hövener

**Affiliations:** 1 Abteilung für Epidemiologie und Gesundheitsmonitoring, Robert Koch-Institut, Gerichtstr. 27, 13347 Berlin, Deutschland; 2 AG 2 Bevölkerungsmedizin und Versorgungsforschung, School of Public Health, Universität Bielefeld, Bielefeld, Deutschland; 3 Sektion Health Equity Studies & Migration, Universitätsklinikum Heidelberg, Heidelberg, Deutschland; 4 Hochschule für angewandte Wissenschaften, Fachbereich Soziale Arbeit, Alice Salomon Hochschule Berlin, Berlin, Deutschland

**Keywords:** Gesundheitsberichterstattung, Datenqualität, Routinedaten, Surveys, Datenverknüpfung, Public health reporting, Data quality, Routine data, Surveys, Data linkage

## Abstract

**Hintergrund:**

Für eine evidenzbasierte Gesundheitspolitik sind aktuelle, repräsentative Daten über alle Bevölkerungsgruppen sowie inklusive Datensysteme unverzichtbar. In der Verfüg- und Verknüpfbarkeit migrationsbezogener Gesundheitsdaten bestehen jedoch erhebliche Lücken. Am Beispiel der psychischen Gesundheit von Kindern und Jugendlichen (KiJu) mit Migrationsgeschichte wird eine Bestandsaufnahme der in Deutschland verfügbaren Datenquellen vorgenommen und deren Nutzbarkeit für die Gesundheitsberichterstattung (GBE) evaluiert.

**Methoden:**

Im Projekt STRONGDATA-Kids wurde ein Mapping von Datenquellen (seit 2000) durchgeführt, die Informationen zur psychischen Gesundheit von KiJu mit Migrationsgeschichte und eine Stichprobengröße von *n* ≥ 1000 aufweisen. Die Datenqualität sowie Zugangs- und Nutzungsbarrieren wurden mittels einer Scorecard anhand von acht Dimensionen bewertet.

**Ergebnisse:**

Es wurden 37 Datenquellen identifiziert. Seit 2019 zeigt sich eine Diversifikation in der Nutzung migrationsbezogener Indikatorik mit Fokus auf Geburtsland (auch der Eltern). Am häufigsten erhoben wurden personale Ressourcen/Resilienz und emotionale- und Verhaltensprobleme. Routinedaten weisen Standardisierung und Zuverlässigkeit auf, Surveys Aktualität und Flexibilität. Die größten Zugangsbarrieren bestehen bei Routinedaten und Surveys auf struktureller Ebene, durch u. a. dezentrale Zuständigkeiten.

**Diskussion:**

Routinedaten und Surveys sind ergänzend unverzichtbar für die GBE. Trotz vielfältigerer migrationsbezogener Indikatoren sind methodische und strukturelle Weiterentwicklungen, etwa standardisierte Indikatorensets, notwendig. Ebenso braucht es diversitätsorientierte Forschungsstrategien und systematische Datenverknüpfungen, um die psychische Gesundheit von KiJu valide darzustellen.

**Zusatzmaterial online:**

Zusätzliche Informationen sind in der Online-Version dieses Artikels (10.1007/s00103-026-04246-2) enthalten.

## Abstract (en)

**Background:**

Up-to-date and representative data across all populations, as well as inclusive data systems, are essential for evidence-based health policy. However, there are considerable gaps in the availability and linkage of migration-related health data. Using the example of the mental health of children and adolescents (ChAd) with a history of migration, this analysis provides an overview of available data sources in Germany and evaluates their usability for public health reporting (PHR).

**Methods:**

Within the STRONGDATA-Kids project, data sources since 2000 were mapped if they included information on the mental health of ChAd with a history of migration and a sample size of *n* ≥ 1000. Data quality and barriers to access and use were assessed using a scorecard comprising eight dimensions.

**Results:**

A total of 37 data sources were identified. Since 2019, a diversification in the use of migration-related indicators can be observed, particularly with regard to country of birth (including that of parents). Personal resources and resilience, as well as emotional and behavioural problems, were most frequently assessed. Routine data show strengths in standardisation and reliability, while surveys ensure timely provision of data and flexibility. The main access barriers to routine data and surveys occur at the structural level, partly due to decentralised responsibilities.

**Discussion:**

Routine data and surveys are complementary and both indispensable for PHR. Despite broader use of migration-related indicators, further methodological and structural developments, such as standardised indicator sets, diversity-oriented research strategies and systematic data linkage are needed to validly represent the mental health of ChAd.

## Einleitung

Deutschland ist geprägt durch eine vielfältige Bevölkerungsstruktur, in der internationale Migration eine wesentliche Rolle spielt. 2023 lag der Anteil der Bevölkerung mit Einwanderungsgeschichte an der Gesamtbevölkerung bei 25,5 % [1], dies entspricht etwa 21,2 Mio. Menschen in Deutschland [2]. Unter Kindern und Jugendlichen (Altersgruppe: 0 bis 18 Jahre) ist dieser Anteil mit 27,8 % höher [1]. Nach Definition der Fachkommission der Bundesregierung zu den Rahmenbedingungen der Integrationsfähigkeit zählen zu den Personen mit Einwanderungsgeschichte jene, die selbst oder deren beide Elternteile seit 1950 nach Deutschland eingewandert sind [3].

Menschen mit Einwanderungsgeschichte sind sehr heterogen und befinden sich in verschiedenen sozialen, rechtlichen und ökonomischen Lebenslagen, die kaum durch starre statistische Kategorien adäquat abgebildet werden können. Die Diversität spiegelt sich in soziodemografischen Merkmalen wie Alter oder Geschlechtsidentität, sozialer Lage oder Chancen und Barrieren zu gesellschaftlicher Teilhabe wider. Die genannten Faktoren sind oft eng miteinander verknüpft, beeinflussen sich gegenseitig und können komplexe Auswirkungen auf die Gesundheit haben [4–7]. Der in der vorliegenden Arbeit genutzte Begriff *Mensch mit Migrationsgeschichte* bezieht sich auf alle Menschen, die einen eigenen oder familiären biografischen Bezug zu Migration und/oder Flucht haben. Er soll dabei nicht als statistische Kategorie im engeren Sinne verstanden werden, sondern vielmehr als konzeptioneller Begriff, der über formale Zuschreibungen – auch durch statistische Kategorien – hinausgeht. Wir verwenden ihn hier als übergreifendes Konstrukt, da in den analysierten Datenquellen durch verschiedene Indikatoren Migrationsbezüge hergestellt werden.

Aus epidemiologischer, entwicklungsbiologischer und -psychologischer Perspektive stellen Kindheit und Jugend, unabhängig von einer etwaigen Migrationsgeschichte, in vielerlei Hinsicht eine sensitive Lebensphase dar, in der Erlebnisse und Expositionen vielfältige Wirkungen auf den späteren Gesundheitsverlauf entfalten können [8–11]. Der Organismus befindet sich in der Entwicklung, was Kinder und Jugendliche besonders empfänglich gegenüber Umwelt- und sozialen Einflüssen macht [10–13]. Auch die emotionale und kognitive Entwicklung wird wesentlich durch die jeweiligen Lebensbedingungen mitgeprägt. Sie kann durch eine unterstützende Sozialwelt gefördert, aber auch durch belastende Lebenserfahrungen negativ beeinflusst werden [14]. Besonders kritisch können traumatische Erlebnisse, wie etwa Fluchterfahrungen oder Ungewissheit über den Aufenthaltsstatus, sowie strukturelle Benachteiligung und familiäre Armut vor, während und nach der Migration wirken. Insbesondere bei fehlenden Unterstützungsangeboten können sie tiefgreifende, lang anhaltende gesundheitliche Folgen haben. Kinder und Jugendliche sind in sozialer Hinsicht besonders schutzbedürftig, da sie in hohem Maße abhängig von Betreuungspersonen und gesellschaftlichen bzw. staatlichen Rahmenbedingungen sind.

Kinder aus Familien mit Migrationsgeschichte können häufiger einen eingeschränkten Zugang zur Gesundheitsversorgung oder zu Präventionsangeboten haben, beispielweise aufgrund von Sprachbarrieren, sozioökonomischen Ungleichheiten oder Diskriminierung und Rassismus [15, 16]. Diese Erfahrungen sind nicht nur sozial belastend, sondern stehen in engem Zusammenhang mit negativen gesundheitlichen Folgen. Empirische Daten aus Deutschland zeigen, dass Kinder und Jugendliche mit Migrationsgeschichte häufiger sowohl von internalisierenden (z. B. Ängstlichkeit, depressive Verstimmungen) als auch externalisierenden (z. B. Aggressivität) psychischen Problemen betroffen sind als Gleichaltrige ohne Migrationsgeschichte, insbesondere wenn eine Fluchterfahrung vorliegt [17, 18].

Zur Identifikation solcher gesundheitlichen Ungleichheiten, allgemein und auch im Bereich psychischer Gesundheit von Kindern und Jugendlichen mit Migrationsgeschichte sowie für eine evidenzbasierte und nachhaltige Gesundheitspolitik, braucht es aktuelle und repräsentative Gesundheitsdaten sowie effektive Datensysteme, die Informationen über alle Bevölkerungsgruppen integrieren. Entscheidend für eine adäquate Stärkung des Gesundheitssystems in Deutschland ist dabei aber auch ein Monitoring der gesundheitlichen Bedarfe und des Zugangs zu Versorgungsangeboten. Die hierfür zur Verfügung stehenden Datensysteme sind bislang klar unzureichend [19, 20]. Migrationsbezogene Merkmale werden uneinheitlich erfasst, was die Vergleichbarkeit zwischen Datensystemen erschwert. Hinzu kommen eine fragmentierte Datenlandschaft und begrenzte Möglichkeiten zur Datenverknüpfung.

Eine Übersichtsarbeit über die Datensysteme in den 53 Ländern der Weltgesundheitsorganisation-(WHO-)Region Europa zeigt eklatante Lücken bei der Erhebung und Integration von Daten zu Migration und Gesundheit, auch in Deutschland [20]. Eine Bestandsaufnahme aus dem Jahr 2019 zu vorhandenen Datenquellen zur gesundheitlichen Lage von Menschen mit Migrationsgeschichte, die vor allem auf die erwachsene Bevölkerung abzielt, dokumentiert ebenfalls eine starke Heterogenität in der Erfassung migrationsbezogener Merkmale [21]. Die Defizite sind umso größer, je stärker die entsprechenden Bevölkerungsgruppen von Marginalisierung betroffen sind, beispielsweise ist die Leistungsfähigkeit von Datensystemen zur Abbildung der gesundheitlichen Lage von Kindern und Jugendlichen mit unsicherem Aufenthaltsstatus besonders limitiert [20].

Da keine einzelne Datenquelle ausreicht, um die Gesundheit von Menschen mit Migrationsgeschichte abzubilden, müssen Datensysteme auf eine Vielzahl unterschiedlicher, im Idealfall miteinander kompatibler, verknüpfbarer oder vergleichbarer Datenquellenarten – wie z. B. Registerdaten, klinische Versorgungsdaten, Befragungsdaten, behördliche und/oder zivilgesellschaftliche Routinedaten – aufbauen, um ein ganzheitliches Bild der gesundheitlichen Lage zeichnen zu können [22]. Die WHO empfiehlt, Datenlücken systematisch zu erfassen und Datensysteme methodisch und strukturell weiterzuentwickeln, um das Gesundheitssystem sowie die zugrunde liegenden Datensysteme nachhaltig zu stärken [23].

Vor diesem Hintergrund ist es Ziel der vorliegenden Arbeit, eine systematische Bestandsaufnahme von Datenquellen durchzuführen, die beispielhaft auf die psychische Gesundheit von Kindern und Jugendlichen mit Migrationsgeschichte fokussiert. Darauf aufbauend erfolgt eine kritische Evaluierung dieser Datenquellen hinsichtlich ihrer Nutzbarkeit für die Gesundheitsberichterstattung (GBE) anhand einer Auswahl etablierter Kriterien zur Bewertung von Gesundheitsinformationssystemen [24]. Dabei werden vorhandene Stärken und Schwächen der Datensysteme sowie bestehende Zugangs- und Nutzungshürden identifiziert, um perspektivisch Handlungsempfehlungen zur Verbesserung der Datenlage in Deutschland abzuleiten. Die Analyse erfolgte im Rahmen des Projekts „STRONGDATA-Kids“, einem Kooperationsprojekt der Universität Bielefeld und des Robert Koch-Instituts, das die Stärkung von Datensystemen zu Migration und Gesundheit und eine nachhaltigere, zielgerichtete Gesundheitsberichterstattung zum Ziel hat.

## Methoden

### Identifikation der Datenquellen.

Die Identifikation der Datenquellen erfolgte in einem 3‑stufigen Rechercheprozess: (1) ausgehend von den Ergebnissen eines systematischen Literaturreviews, das im Rahmen des STRONGDATA-Kids-Projektes zur psychischen Gesundheit von Kindern und Jugendlichen mit Migrationsgeschichte in Deutschland durchgeführt wurde, (2) durch Hinweise von Expert:innen aus dem Forschungsfeld sowie einer themenverwandten Publikation aus dem Jahr 2019 [21] und (3) durch eine erweiterte webbasierte Recherche über die Datensammlung der GBE-Bund www.gbe-bund.de [21]. Die Zusammenstellung der Schuleingangsuntersuchungen (SEU) in Deutschland hingegen erfolgte über eine webbasierte Recherche der Eltern- bzw. Anamnesebögen der einzelnen Bundesländer.

### Ein- und Ausschlusskriterien.

Datenquellen wurden in das Mapping einbezogen, wenn sie (1) Daten seit dem Jahr 2000 in Deutschland, (2) mindestens ein Outcome zur Beschreibung der psychischen Kinder- und Jugendgesundheit und (3) eine Mindeststichprobengröße von *n* = 1000 aufwiesen. Zusätzlich mussten sie (4) mindestens einen der folgenden migrationsbezogenen Indikatoren enthalten: (a) Geburtsland der Kinder/Jugendlichen, (b) Geburtsland von mindestens einem Elternteil, (c) die Staatsangehörigkeit der Kinder/Jugendlichen, (d) die Staatsangehörigkeit von mindestens einem Elternteil, (e) das Einreisejahr, (f) den Aufenthaltsstatus, (e) sprachrelevante Merkmale (zu Hause gesprochene Sprache und/oder selbsteingeschätzte Deutschkenntnisse).

### Mapping.

Im ersten Schritt wurde zwischen Routinedaten und Surveys unterschieden, gefolgt von der Unterscheidung nach quer- bzw. längsschnittlicher Datenerhebung. Im Anschluss an die Identifikation und Kategorisierung relevanter Datenquellen wurden diese im Vieraugenprinzip anhand ausgewählter Parameter (Tab. A1 im Onlinematerial) beschrieben. Die Auswahl dieser Parameter erfolgte unter Einbeziehung fachnaher Akteur:innen über einen Online-Workshop und orientierte sich an ihrer Relevanz für die Bewertung der Nutzbarkeit der Datenquellen für die GBE. Erfasst wurden dabei sowohl allgemeine Informationen als auch migrationsbezogene Merkmale sowie Angaben zu psychischen Gesundheitsoutcomes.

### Evaluierung per Scorecard.

Die Evaluierung der identifizierten Datenquellen hinsichtlich ihrer Qualität, Zugänglich- und Nutzbarkeit erfolgte anhand einer im Projekt entwickelten Scorecard (Abb. A1 im Onlinematerial), die etablierte Kriterien zur Bewertung von Gesundheitsinformationssystemen [24] zusammenbringt. Um praxisnahe und interdisziplinäre Perspektiven in die Entwicklung der Scorecard einzubeziehen, wurden geeignete Kriterien unter Beteiligung von Stakeholdern aus Wissenschaft, Politik und Praxis identifiziert und angepasst.

Konkret umfasst sie die Dimensionen Genauigkeit, Konformität, Konsistenz, Zuverlässigkeit und Aktualität, zentrale Qualitätsmerkmale, die für die wissenschaftliche Nutzbarkeit von Gesundheitsdaten entscheidend sind [24]. Darüber hinaus wurden 3 weitere Dimensionen aufgenommen, um potenzielle Zugangs- und Nutzungsbarrieren zu erfassen: rechtliche, finanzielle und strukturelle Barrieren. Da konkret nach der Abwesenheit von Barrieren gefragt wird (z. B. „Die Daten können ohne rechtliche Barrieren abgerufen und genutzt werden“), sind hierbei niedrige Scorewerte gleichbedeutend mit höheren Barrieren.

Die Evaluierung durch das Projektteam erfolgte in 3 Schritten: Zunächst wurden grundlegende Informationen (z. B. Name der Datenquelle, Datenumfang und geografische Verteilung) zu den Datenquellen extrahiert. Danach erfolgte die Bewertung der verschiedenen Dimensionen anhand einer 5‑stufigen Likert-Skala (1 = „stimme überhaupt nicht zu“ bis 5 = „stimme voll und ganz zu“ bzw. 0 = „nicht zutreffend/unklar“) durch 2 Projektmitarbeitende unabhängig voneinander qualitativ auf Basis der Kernfragen. Ein höherer Skalenwert wurde vergeben, wenn die Mehrheit der Kernfragen positiv beantwortet wurde, ein niedrigerer bei überwiegend negativen Einschätzungen. Bei gemischten Befunden wurde die Mittelkategorie gewählt, bei unzureichender Informationslage wurde 0 („unklar“/„nicht zutreffend“) vergeben. Zur Bestimmung der Interrater-Reliabilität wurde der Spearman-Rangkorrelationskoeffizient ρ getrennt je Dimension sowie insgesamt über alle Dimensionen hinweg berechnet. In einem letzten Schritt wurden die im Vieraugenprinzip evaluierten Datenquellen im Projektteam konsentiert. Nach Bewertung aller Datenquellen wurden die Ergebnisse analysiert, um Stärken, Schwächen sowie Nutzungs- und Zugangsbarrieren zu identifizieren. Zur Ergänzung fehlender Informationen sowie zur Validierung der vom Projektteam vorgenommenen Scorecard-Evaluierungen wurden die Datenhaltenden kontaktiert. Wenn Rückmeldung dazu durch die Datenhaltenden erfolgte, fand ein direkter Austausch statt.

## Ergebnisse

### Allgemeine Beschreibung der Datenquellen

Es wurden insgesamt 48 Datenquellen zur Gesundheit von Kindern und Jugendlichen mit Migrationsgeschichte identifiziert, 37 erfüllten die Einschlusskriterien und wurden in das Mapping aufgenommen, darunter 16 Datenquellen aus den Schuleingangsuntersuchungen (SEU) aller Bundesländer, die im Folgenden gesondert betrachtet werden. Zentrale Informationen zu den Datenquellen (exklusive SEU) sind in Tab. 1 dargestellt.

**Tab. 1:** Ausgewählte Charakteristika der in das Mapping eingeschlossenen Datenquellen, ohne Schuleingangsuntersuchungen* n* = 21

Name der Datenquelle	Datentyp	Design	Räumlichkeit	Stichprobe	Zeitraum	Migrationsindikatorik							
						GebL	GebLE	StA	StAE	EinJ	AufS	Deu	Spra
AID:A – Aufwachsen in Deutschland: Alltagswelten	Survey	Längsschnitt	Bundesweit	> 10.000	Jährlich (seit 2009)	x	x	–	–	–	–	–	x
AOK-Familienstudie 2022	Survey	Querschnitt	Bundesweit	5000–10.000	2022	x	x	–	–	–	–	–	x
BELLA – BEfragung zum seeLischen WohLbefinden und VerhAlten	Survey	Längsschnitt	Bundesweit	< 5000	2003–2006 2014–2017	x	x	x	x	x	–	–	x
CILS4EU – Children of Immigrants Longitudinal Survey in Four European Countries	Survey	Längsschnitt	Multinational	> 10.000	2010–2022	x	x	x	x	x	–	x	x
COPSY-Studie (Corona und Psyche)	Survey	Längsschnitt	Bundesweit	< 5000	Jährlich (Seit 2020)	x	x	x	x	–	–	–	x
EU-SILC – EU Statistics on Income and Living Conditions	Survey	Querschnitt	Multinational	> 10.000	Jährlich (seit 2003)	x	x	x	x	x	–	–	x
GKV-Daten* – Abrechnungsdaten der gesetzlichen Krankenversicherung (GKV)	Routine	Querschnitt	Bundesweit	> 10.000	Jährlich	–	–	x	–	–	–	–	–
Gesundheitsmonitoring Einheiten (GME)	Survey	Querschnitt	Kommunal	> 10.000	Jährlich (seit 2004)	x	x	–	–	–	–	–	x
HBSC – Health Behaviour in School-aged Children	Survey	Längsschnitt	Bundesweit	> 10.000	1993–2022	x	x	–	x	–	–	–	x
IAB-BAMF-SOEP-Befragung von Geflüchteten	Survey	Längsschnitt	Bundesweit	> 10.000	Jährlich (seit 2016)	x	x	x	x	x	x	x	x
IAB-BiB/FReDA-BAMF-SOEP-Befragung	Survey	Längsschnitt	Bundesweit	> 10.000	2022–2023	x	x	–	x	x	–	–	–
IAB-SOEP Migrationsstichprobe	Survey	Längsschnitt	Bundesweit	> 10.000	Jährlich (seit 2013)	x	x	x	x	x	x	x	x
IDEFICS – Identification and prevention of Dietary- and lifestyle-induced health EFfects In Children and infantS	Survey	Längsschnitt	Multinational	< 5000	2006–2012	x	x	x	–	–	–	–	–
Integrate ADHD	Survey	Querschnitt	Bundesweit	5000–10.000	2021–2024	x	x	x	x	x	–	–	x
KIDA – Kindergesundheit in Deutschland aktuell	Survey	Querschnitt	Bundesweit	5000–10.000	2022–2023	x	x	–	x	x	–	–	–
KiGGS – Studie zur Gesundheit von Kindern und Jugendlichen in Deutschland	Survey	Querschnitt/Längsschnitt	Bundesweit	> 10.000	2003–2006 2009–2012 2014–2017	x	x	x	x	x	x	–	x
Mikrozensus	Routine	Querschnitt	Bundesweit	> 10.000	Jährlich (seit 1957)	x	x	x	x	x	–	–	x
NEPS – Nationales Bildungspanel	Survey	Längsschnitt	Bundesweit	> 10.000	Jährlich (seit 2009)	x	x	x	x	x	x	x	x
Pairfam – Deutscher Beziehungs- und Familienpanel	Survey	Längsschnitt	Bundesweit	> 10.000	2008–2022	–	x	–	x	–	–	x	–
Pri.Care – Health and primary-care sentinel surveillance in reception- and accommodation-centres for asylum-seekers in Germany	Routine	Querschnitt/Längsschnitt	Regional	> 10.000	2016–2020	–	–	x	–	–	x	–	x
ReGES – Refugees in the German Educational System Projekt	Survey	Längsschnitt	Regional	< 5000	2016–2021	x	x	–	x	x	x	x	x

* Die GKV-Daten umfassen Abrechnungsdaten der AOK (*WIdO* Wissenschaftliches Institut der AOK), Barmer (Institut für Gesundheitssystemforschung), Techniker Krankenkasse (Institut für Versorgungsforschung)

*Migrationsindikatorik*: *GebL* Geburtsland der Kinder/Jugendlichen, *GebLE* Geburtsland von mindestens einem Elternteil, *StA* Staatsangehörigkeit der Kinder/Jugendlichen, *StAE* Staatsangehörigkeit von mindestens einem Elternteil, *EinJ* Einreisejahr, *AufS* Aufenthaltsstatus, *Deu* selbsteingeschätzte Deutschkenntnisse, *Spra* zu Hause gesprochene Sprache, *IAB* Institut für Arbeitsmarkt- und Berufsforschung, *BiB* Bundesinstitut für Bevölkerungsforschung, *FReDA* Family Research and Demographic Analysis, *BAMF* Bundesamt für Migration und Flüchtlinge, *SOEP* Sozio-oekonomisches Panel

Es wurden 3 Routinedatenquellen und 18 Surveys – darunter 7 Quer- und 12 Längsschnittstudien – eingeschlossen sowie 2 Datenquellen, die sowohl Längs- als auch Querschnitterhebungen in sich vereinen. Hinsichtlich der räumlichen Ebene zeigt sich folgendes Bild: Auf multinationaler Ebene gibt es 3 Surveys mit Quer- und Längsschnittdesign mit separat berichteten Daten für Deutschland. Die bundesweiten Quellen setzen sich überwiegend aus Surveys mit Längs- (*n* = 10) bzw. Querschnittdesign (*n* = 5) zusammen, ergänzt durch 2 Routinedatenquellen mit Querschnittdesign. Auf regionaler Ebene gibt es 2 Datenquellen, die sowohl Routine- als auch Surveydaten erheben. Auf kommunaler Ebene liegt neben den SEU der einzelnen Bundesländer (siehe Abschnitt Schuleingangsuntersuchungen) nur eine Surveydatenquelle vor.

**Abb. 1:**
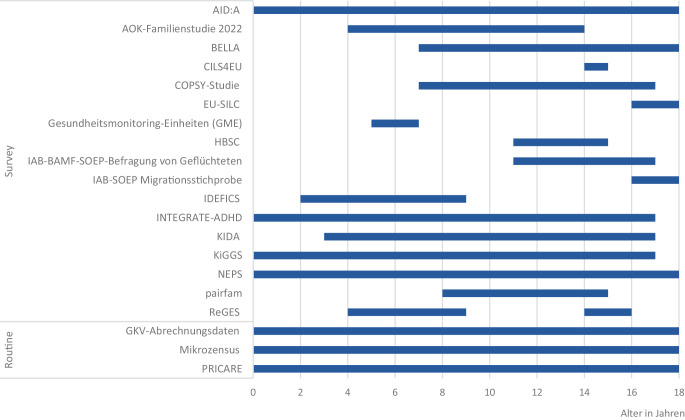
Altersverteilung von Kindern und Jugendlichen in den Datenquellen, ohne Schuleingangsuntersuchungen (*n* = 20). Die Datenquelle IAB-BiB/FReDA-BAMF-SOEP-Befragung wurde in der Abbildung nicht berücksichtigt, da sie ausschließlich die Erwachsenenbevölkerung adressiert. Sie enthält allerdings auch gesundheitsrelevante Informationen zu deren Kindern und wurde daher in die Gesamtanalysen mit einbezogen

Die Stichprobengrößen variieren zwischen *n* = 1040 und weitestgehenden Vollerhebungen im Rahmen der SEU. Mittelgroße bis große Datenquellen umfassen mehrere Tausend Kinder und Jugendliche in bundesweiten Surveys, wie der KiGGS-Studie [25]. Die größten Datenquellen sind fortlaufende Panel- und Kohortenstudien wie das Nationale Bildungspanel (NEPS) mit über 80.000 Teilnehmenden (einschließlich Kinder‑, Jugend- und Erwachsenenkohorten) oder der Pri.Care-Datensatz, in dem anonymisierte Daten von mehr als 200.000 (darunter auch minderjährigen) Patient:innen erfasst werden [26, 27]. Die Mehrheit der eingeschlossenen Datenquellen erfasst Kinder und Jugendliche im Alter von 7 bis 17 Jahren (Abb. 1).

### Verwendete Migrationsindikatorik

**Abb. 2:**
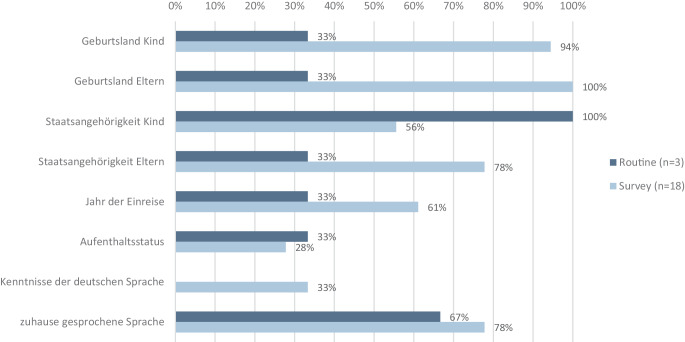
Erfassung verschiedener migrationsbezogener Variablen. Relative Häufigkeit in *n* = 21 Datenquellen (ohne Schuleingangsuntersuchungen)

In den identifizierten Datenquellen (exklusive SEU) werden migrationsbezogene Indikatoren mit unterschiedlicher Häufigkeit erfasst: am häufigsten das Geburtsland des Kindes (Surveys: 94 %, Routine: 33 %) sowie das der Eltern (Surveys: 100 %, Routine: 33 %) und die zu Hause gesprochene Sprache (Surveys: 78 %, Routine: 67 %; Abb. 2). Ebenfalls regelmäßig berücksichtigt werden die Staatsangehörigkeit des Kindes (Surveys: 56 %, Routine: 100 %) und der Eltern (Surveys: 78 %, Routine: 33 %). Seltener finden sich Angaben zu Deutschkenntnissen (Surveys: 33 %) und dem Aufenthaltsstatus (Surveys: 28 %, Routine: 33 %).

Auch bei der Kombination migrationsbezogener Indikatoren (inklusive SEU; Abb. A2 im Onlinematerial) zeigt sich ein Muster: In Routinedaten werden besonders häufig das Geburtsland des Kindes und die Geburtsländer der Eltern (84 %) zusammen erfasst. Andere Merkmale, wie das Jahr der Einreise oder die zu Hause gesprochene Sprache, werden nur in einem kleineren Teil der Datenquellen gemeinsam erhoben. Angaben zum Aufenthaltsstatus in Kombination mit anderen Indikatoren fehlen bei Routinedaten, mit Ausnahme des Pri.Care-Datensatzes [27]. Surveys hingegen kombinieren deutlich häufiger mehrere Merkmale, beispielsweise Geburtsland der Eltern mit Staatsangehörigkeit der Eltern (78 %), Jahr der Einreise (61 %) und zu Hause gesprochener Sprache (78 %), wobei der Aufenthaltsstatus in Kombination mit anderen Merkmalen ebenfalls nur in wenigen Surveys erfasst wird.

### Erfasste Indikatoren der psychischen Gesundheit

**Abb. 3:**
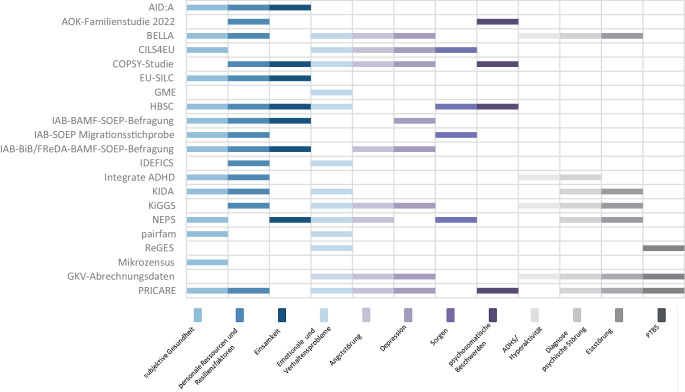
Psychische Gesundheitsoutcomes eingeschlossener Datenquellen, ohne Schuleingangsuntersuchungen (*n* = 21)

In Bezug auf die psychische Gesundheit werden besonders häufig erfasst: subjektive Gesundheit (als unspezifischer bzw. globaler Indikator für psychisches Wohlbefinden; 66,7 %), personale Ressourcen und Resilienzfaktoren (66,7 %) sowie emotionale- und Verhaltensprobleme (61,9 %; Abb. 3). Die Aufmerksamkeits‑/Hyperaktivitätsstörung (ADHS), Essstörungen oder posttraumatische Belastungsstörung (PTBS) werden hingegen seltener berücksichtigt.

### Evaluierung mittels Scorecard

**Abb. 4:**
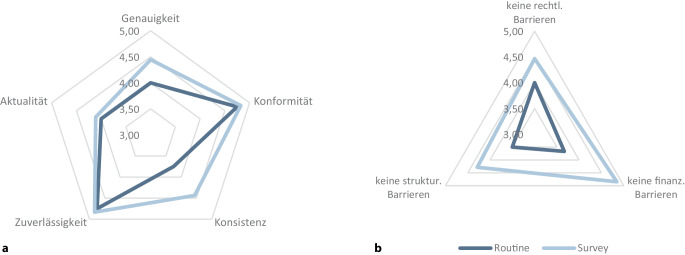
Bewertung der Datenquellen nach Scorecard-Dimensionen (Routinedaten vs. Surveys, Median, ohne Schuleingangsuntersuchungen *n* = 21) in Hinblick auf **a** Qualität und **b** Zugangs- und Nutzungsbarrieren. Hinweis: Die Skalen beginnen ab dem Wert 3,0

Abb. 4 zeigt die Ergebnisse der Evaluierung mittels Scorecard. Der Spearman-Rangkorrelationskoeffizient beträgt insgesamt ρ = 0,69 und weist damit auf eine hohe Übereinstimmung zwischen den Bewertungen der beiden Projektmitarbeitenden hin. Bei den Routinedaten liegen die Werte, insbesondere bei Konformität (Median: 4,8) und Zuverlässigkeit (Median: 4,8) der Datenquellen, sehr hoch, was auf standardisierte Datenerhebungsprozesse und gut dokumentierte Methoden zurückzuführen ist. Hingegen zeigen sich für die Konsistenz (Median: 3,8), also die Variabilität der Indikatoren, und auch die Aktualität (Median: 4,0) der Datenquellen eher niedrigere Werte. Surveys erreichen ebenso die höchsten Werte bei Konformität (Median: 4,8) und Zuverlässigkeit (Median: 4,8). Bei der Konsistenz (Median: 4,4) und der Aktualität (4,1) liegen die Medianwerte über jenen der Routinedaten. Damit werden Surveys vor allem als aktueller und variabler eingeschätzt, während Routinedaten ihre Stärken in formaler Genauigkeit und Standardisierung haben.

Hinsichtlich der Zugangs- und Nutzungsbedingungen werden strukturelle Barrieren für Routinedaten am stärksten^1^ als Zugangshindernis wahrgenommen (Median: 3,5). Diese hängen vor allem mit dezentralen Zuständigkeiten (z. B. bei den SEU) und komplexen Antragsverfahren zusammen. Finanzielle (Median: 3,7) sowie rechtliche Barrieren (Median: 4,0) werden ebenfalls häufiger als Hindernis im Zugang zu Routinedaten wahrgenommen im Vergleich zu den Surveys. Darunter fallen beispielsweise strengere Datenschutzregelungen, die die Nutzung und Weitergabe von Gesundheitsdaten für wissenschaftliche Zwecke einschränken.

Auch bei Surveys stellen strukturelle Barrieren (Median: 4,3) das größte Hindernis dar, sind aber weniger ausgeprägt als bei Routinedaten. Rechtliche (Median: 4,5) und finanzielle Barrieren (Median: 4,8) werden im Vergleich zu Routinedatenquellen als weniger ausgeprägt wahrgenommen, was auf geringere Zugangshürden und meist kostenfreie Nutzung für wissenschaftliche Zwecke zurückzuführen ist. Eine vollständige Übersicht der Bewertungen findet sich im Anhang (Tab. A2 im Onlinematerial).

### Schuleingangsuntersuchungen

Die SEU der einzelnen Bundesländer in Deutschland stellen eine wichtige Routinedatenquelle mit kommunaler Abdeckung dar. Sie erfassen alle schulpflichtigen Kinder im Alter von 5 bis 7 Jahren und haben zum Ziel, gesundheits- bzw. entwicklungsspezifische Aussagen über jedes Kind vor Schulbeginn treffen zu können. Die SEU werden bundesweit von den Kinder- und Jugenddiensten der örtlichen Gesundheitsämter durchgeführt [28]. Aufgrund des föderalen Systems in Deutschland erfolgt ihre Durchführung nicht einheitlich.

Alle Bundesländer berücksichtigen mehr als einen migrationsbezogenen Indikator (Tab. 2). Am häufigsten erfasst werden das Geburtsland der Eltern (*n* = 16), das Geburtsland des Kindes (*n* = 15) und die Staatsangehörigkeit der Eltern (*n* = 13). Weniger systematisch erfasst werden das Einreisejahr und sprachbezogene Merkmale. Der Aufenthaltsstatus wird über die Eltern- bzw. Anamnesefragebögen nicht erfasst, eine Fluchterfahrung lässt sich jedoch über sogenannte Seiteneinsteigeruntersuchungen, zumindest in einzelnen Bundesländern wie Nordrhein-Westfalen oder Hessen, approximieren [29]. Grundsätzlich zeigt sich, dass die Erfassung migrationsbezogener Indikatoren sehr fragmentiert und uneinheitlich, auch innerhalb einzelner Bundesländer, vorgenommen wird. Hinsichtlich der Gesundheitsoutcomes werden am häufigsten emotionale und Verhaltensprobleme sowie ADHS erfasst, wenn auch nicht flächendeckend in allen Bundesländern. Einige spezifische Outcomes (z. B. Angststörungen und (Symptome von) Depressionen) werden nur vereinzelt bei den SEU erhoben.

**Tab. 2:** Schuleingangsuntersuchungen der Bundesländer stratifiziert nach verwendeter migrationsbezogener Indikatorik und psychischen Gesundheitsoutcomes (*n* = 16)

Name der Datenquelle	Migrationsindikatorik								Psychische Gesundheitsoutcomes											
	GebL	GebLE	StA	StAE	EinJ	AufS	Deu	Spra	SubjG	PersRes	Eins	EVP	Angst	Depr	Sorg	PsySom	ADHS	DiagPS	EssST	PTBS
Baden-Württemberg	x	x	–	x	–	–	–	x	–	–	–	–	–	–	x	–	–	–	–	–
Bayern*	x	x	x	–	–	–	x	x	–	–	–	x	–	x	–	–	x	–	–	–
Berlin	x	x	–	x	x	–	x	x	–	–	–	–	–	–	x	–	–	–	–	–
Brandenburg*	x	x	x	x	–	–	–	x	–	–	–	x	–	–	–	–	–	x	–	–
Bremen	x	x	–	x	–	–	x	–	–	–	–	x	–	–	–	–	–	–	–	–
Hamburg	x	x	–	x	–	–	–	–	–	–	–	x	–	–	–	–	–	–	–	–
Hessen	x	x	x	x	x	–	–	–	–	–	–	x	x	–	–	–	x	–	–	–
Mecklenburg-Vorpommern*	x	x	x	–	–	–	–	–	–	–	–	x	x	–	–	–	x	–	–	–
Niedersachsen	x	x	x	x	–	–	–	x	–	–	–	–	–	–	–	–	x	–	–	–
Nordrhein-Westfalen*	x	x	x	x	x	–	–	x	–	–	–	x	–	–	–	–	x	–	–	–
Rheinland-Pfalz*	x	x	–	x	–	–	–	x	–	–	–	x	–	–	–	–	x	x	–	–
Saarland*	x	x	–	x	x	–	x	–	–	–	–	x	–	–	–	–	x	x	–	–
Sachsen*	x	x	–	x	–	–	–	–	–	–	–	x	–	–	x	–	–	–	–	–
Sachsen-Anhalt	x	x	–	x	–	–	–	–	–	–	–	x	–	–	–	–	–	–	–	–
Schleswig-Holstein	–	x	–	–	–	–	–	x	–	–	–	x	–	–	–	–	x	–	–	–
Thüringen*	x	x	x	x	–	–	–	x	–	–	–	x	–	–	–	–	–	–	–	–

* Uneinheitliche Erfassung innerhalb des Bundeslandes

*Migrationsindikatorik*: *GebL* Geburtsland der Kinder/Jugendlichen, *GebLE* Geburtsland von mindestens einem Elternteil, *StA* Staatsangehörigkeit der Kinder/Jugendlichen, *StAE* Staatsangehörigkeit von mindestens einem Elternteil, *EinJ* Einreisejahr, *AufS* Aufenthaltsstatus, *Deu* selbsteingeschätzte Deutschkenntnisse, *Spra* zu Hause gesprochene Sprache; *psychische Gesundheitsoutcomes: subjG* subjektive Gesundheit, *persRes* personale Ressourcen und Resilienzfaktoren, *Eins* Einsamkeit, *EVP* emotionale und Verhaltensprobleme, *Angst* Angst, *Depr* Depression, *Sorg* Sorgen, *psySom* psychosomatische Beschwerden, *ADHS* ADHS, *diagPS* Diagnose psychische Störung, *essST* Essstörung, *PTBS* PTBS

## Diskussion

### Datenquellen und Indikatoren

Ziel der vorliegenden Analyse war es, verfügbare Datenquellen zur psychischen Gesundheit von Kindern und Jugendlichen mit Migrationsgeschichte in Deutschland systematisch zu erfassen und hinsichtlich ihrer Nutzbarkeit für die GBE zu bewerten. Insgesamt konnten 37 Datenquellen eingeschlossen werden, einschließlich der SEU der 16 Bundesländer. Zentral hervorzuheben ist, dass sich seit 2019 die migrationsbezogene Indikatorik diversifiziert hat, insbesondere mit Fokus auf Geburtsland (auch der Eltern) und sprachbezogene Merkmale, und dass personale Ressourcen/Resilienz sowie emotionale und Verhaltensprobleme am häufigsten erfasst werden. Routinedaten überzeugen durch Standardisierung, Surveys durch Aktualität und Flexibilität, während Zugangs- und Nutzungsbarrieren insgesamt vor allem auf struktureller Ebene bestehen.

Eine vergleichbare Bestandsaufnahme liegt für den deutschsprachigen Raum bislang nicht vor. Lediglich 2 systematische Übersichtsarbeiten [20, 21] bieten eine Vergleichsgrundlage im Hinblick auf die Erfassung von Datenquellen bzw. migrationsbezogener Indikatorik, berücksichtigen allerdings vorwiegend die erwachsene Bevölkerung, fokussieren psychische Gesundheitsoutcomes gar nicht und analysieren Datensysteme und Zugangsbarrieren nicht systematisch. Unsere Analyse erweitert diesen Zugang, indem sie speziell Kinder und Jugendliche in den Fokus nimmt und sowohl Datensystemkategorien, Migrationsindikatorik, psychische Gesundheitsoutcomes als auch Nutzbarkeit evaluiert. Während 2019 vor allem die Staatsangehörigkeit der primär genutzte migrationsbezogene Indikator war, sind es im Feld der Kinder und Jugendlichen eher das Geburtsland (inkl. der Eltern) oder sprachbezogene Merkmale. Darüber hinaus werden vielfach mehrere Indikatoren verwendet und miteinander kombiniert.

Auch wenn Routinedaten im Vergleich zu Surveys quantitativ weiterhin eine geringere Varianz an migrationsbezogenen Indikatoren aufweisen, wird der (teils zugeschriebene) Migrationsstatus heute differenzierter erfasst als noch vor wenigen Jahren. Diese Entwicklung ist zwar ein wichtiger Fortschritt, er bleibt jedoch begrenzt, da ohne die Berücksichtigung der sozialen Lage, von Diskriminierungserfahrungen und strukturellen Ausschlüssen oder Ressourcen die Heterogenität von Menschen mit Migrationsgeschichte nicht adäquat widergespiegelt werden kann. Positiv anzuerkennen ist dennoch, dass nicht ausschließlich auf Einzelindikatoren zurückgegriffen, sondern insgesamt eine größere Vielfalt an Indikatoren genutzt wird. Gleichzeitig erschwert der geringe Standardisierungsgrad der Migrationsindikatorik die Vergleichbarkeit der Daten über Datenquellen hinweg.

Im Hinblick auf die Schuleingangsuntersuchungen lässt sich ferner feststellen, dass eine einheitliche Operationalisierung und Erfassung migrationsbezogener Indikatorik auf Bundesebene weiterhin fehlt. Dies stellt vor dem Hintergrund eines wachsenden Bedarfs an vergleichbaren Daten, vor allem im Bereich Migration und Gesundheit, ein wesentliches Defizit dar. In Bezug auf die Erfassung psychischer Gesundheitsoutcomes über alle Datenquellen hinweg zeigt sich ebenfalls ein fragmentiertes Bild hinsichtlich Quantität und Varianz. Entwicklungsbezogene Merkmale wie emotionale und Verhaltensprobleme sowie Ressourcen und Resilienzfaktoren erweisen sich dabei als zentrale Dimensionen der Kinder- und Jugendgesundheit. Dieses Ergebnis findet sich auch in einem Review zur psychischen Gesundheit von Kindern und Jugendlichen in der Allgemeinbevölkerung in Deutschland [30].

Unsere Analyse verdeutlicht zudem, dass regionale Datenquellen vergleichsweise selten sind und sich überwiegend auf die SEU der Bundesländer beschränken. Ein möglicher Grund könnte in den vorab definierten Einschlusskriterien liegen, insbesondere in der festgelegten Mindeststichprobengröße von *n* = 1000.

### Qualität der Datenquellen

In Bezug auf die Datenquellenqualität zeigen sich Unterschiede zwischen Routine- und Surveydaten. So weisen die eingeschlossenen Routinedaten eine hohe Standardisierung, regelmäßige Erhebung und breite räumliche Abdeckung auf, gleichzeitig sind sie meist ausschließlich auf Krankheitsdiagnosen fokussiert. Dieses Bild deckt sich mit anderen Analysen die die Nutzbarkeit von Routinedaten für die Public-Health-Forschung bewerten [31, 32]. Sie verweisen darauf, dass Routinedaten in der Regel kaum psychosoziale und kontextuelle Umweltfaktoren wie die soziale Lage berücksichtigen. Dieses Ergebnis wird durch unsere Bestandsaufnahme bestätigt.

Hinzu kommt, dass bei Routinedaten sowohl methodische Herausforderungen als auch mögliche Unvollständigkeiten der Daten häufig nicht prospektiv berücksichtigt werden können [32]. Bevölkerungsbasierte Surveys ermöglichen hingegen eine breitere Abbildung der gesundheitlichen Lage, nutzen etablierte Messinstrumente und können teilweise durch Längsschnittdesigns auch zeitliche Vergleiche und die Beobachtung von Trends ermöglichen. Sie sind jedoch meist ressourcenintensiv und weisen mitunter Verzerrungen auf, gerade wenn eine diversitätsbezogene Forschungsstrategie nicht mitgedacht wird.

### Datenzugang und -nutzung

Auch beim Datenzugang zeigen sich in unseren Analysen Unterschiede. Der Zugang zu Routinedaten ist, im Vergleich zu Surveydaten, stärker durch rechtliche Barrieren eingeschränkt, da strenge Datenschutzauflagen und komplexere Genehmigungsverfahren gelten. Diese Hürden können zwar die Datennutzung erschweren, dienen jedoch zugleich dem Schutz besonders sensibler Dateninformationen. Hinzu kommen strukturelle Barrieren, etwa durch heterogene Zuständigkeiten bei den Krankenkassen oder den Gesundheitsämtern. Um sowohl den Datenschutz zu gewährleisten als auch die wissenschaftliche Nutzung zu ermöglichen, wäre eine ausgewogene Gestaltung solcher Regelungen zentral. Auch entstehen finanzielle Barrieren, etwa durch Kosten für Datenaufbereitung und -bereitstellung.

Bei Surveydaten bestehen im Hinblick auf den Zugang geringere Hürden: Rechtliche Anforderungen beschränken sich meist auf Nutzungsverträge oder Ethikvoten, finanzielle Barrieren sind selten und strukturelle Barrieren bestehen in der Regel primär in Form von niedrigschwelligeren Antragsverfahren. Einschränkungen ergeben sich bei einzelnen Surveys allerdings auch, etwa durch Sperrfristen, eingeschränkte Verfügbarkeit oder die Notwendigkeit des Vor-Ort-Zugangs in sicheren Datenzentren. Insgesamt zeigt sich jedoch, dass Surveydaten im Bereich der psychischen Gesundheit von Kindern und Jugendlichen mit Migrationsgeschichte für die wissenschaftliche Nutzung leichter zugänglich und inhaltlich flexibler einsetzbar sind als Routinedaten.

### Stärken und Limitationen

Diese Analyse erhebt keinen Anspruch auf Vollständigkeit. Eingeschlossene Datenquellen wurden über ein vorrangegangenes systematisches Literaturreview, die Datensammlung der GBE des Bundes sowie Hinweise durch Expert:innen identifiziert. Vor dem Hintergrund, dass eingeschlossene Datenquellen die Bevölkerung in Deutschland betrachten und eine Mindeststichprobengröße von *n* = 1000 haben mussten, wurden kleinere, qualitative und internationale Studien ohne separat berichtete Daten für Deutschland nicht berücksichtigt. Qualitative Erhebungen, die möglicherweise auch Daten zu Subgruppen wie geflüchtete Kinder und Jugendliche umfassen, konnten somit nicht einbezogen werden. Zudem wurde nicht systematisch erfasst, in welchen Sprachen die Erhebungen durchgeführt wurden. Dies ist insbesondere im Kontext von Kindern und Jugendlichen mit Migrationsgeschichte relevant, da sprachliche Barrieren die Teilnahme beeinflussen können. Gleichwohl bietet die Übersicht eine wertvolle Grundlage, um verfügbare Datenquellen im Themenbereich psychische Gesundheit von Kindern und Jugendlichen mit Migrationsgeschichte gezielt zu identifizieren und hinsichtlich ihrer Stärken und Schwächen zu evaluieren und für zukünftige Forschungszwecke nutzbar zu machen.

## Fazit

Die Bestandsaufnahme und Evaluierung konnten zeigen, dass sowohl Routinedaten als auch Surveys für die GBE unverzichtbare, sich ergänzende Informationsquellen mit unterschiedlichen Stärken und Schwächen sind. Eine diversitätsorientierte Erhebungsstrategie, die Menschen mit Migrationsgeschichte systematisch mitdenkt, stellt eine zentrale Voraussetzung dar, um gesundheitliche Ungleichheiten sichtbar zu machen. Ergänzend ist eine Verknüpfbarkeit unterschiedlicher Primär- und Sekundärdaten nötig, um vorhandene Daten zusammenzuführen und Bedarfe so besser aufzeigen zu können. Hierfür müssen jedoch gesetzliche Rahmenbedingungen geschaffen werden, die die Verknüpfung und disaggregierte Analysen besonders schutzbedürftiger Daten für Gesundheitsforschung und eine wissenschaftlich fundierte GBE ermöglichen. Zudem sollten Forschungsdatenzentren sowie wissenschaftliche Einrichtungen die dafür notwendigen technisch-praktischen Strukturen aufbauen bzw. in bestehende Strukturen integrieren. Darüber hinaus bedarf es einer methodischen Weiterentwicklung, etwa durch standardisierte Indikatorensets, die migrations- und diversitätsbezogene Merkmale systematisch erfassen. Zudem sollten sowohl das Mapping als auch die Scorecard-Evaluation von Datensystemen auch zu anderen Alters- und Bevölkerungsgruppen und Gesundheitsoutcomes in regelmäßigen Intervallen durchgeführt werden. Verbunden mit den Erkenntnissen aus den jeweiligen Datensystemen, deren Ergebnisse zentral gebündelt und für Forschende öffentlich zugänglich gemacht werden sollten, ließe sich so ein ganzheitliches und differenziertes Bild der gesundheitlichen Lage von Menschen mit Migrationsgeschichte in Deutschland abbilden, das als Grundlage für die Entwicklung von konkreten Handlungsempfehlungen dienen kann, mit dem Ziel, eine bessere Gesundheitspolitik für alle zu ermöglichen.

## Supplementary Information

Onlinematerial: Zusätzliche Abbildungen und Tabellen

## Data Availability

Die für die Studie zugrunde liegenden Daten umfassen eine systematische Übersicht der identifizierten Datenquellen sowie die im Rahmen der Scorecard vorgenommenen Evaluierungen. Da diese Daten weitgehend den im Artikel berichteten Ergebnissen entsprechen, stehen sie auf Anfrage zu wissenschaftlichen Zwecken bei der Korrespondenzautorin zur Verfügung.

## Footnotes

1 Da die als negativ zu evaluierenden Dimensionen der Zugangs- und Nutzungsbarrieren (*rechtliche, finanzielle, strukturelle Hindernisse*) auf derselben Likert-Skala bewertet wurden wie die als positiv zu evaluierenden Dimensionen *Genauigkeit, Konformität, Konsistenz, Zuverlässigkeit *und *Aktualität,* sind an dieser Stelle niedrige Medianwerte gleichbedeutend mit höheren Barrieren.
