# Supplementary material for: Inventory and evaluation of data sources on the mental health of children and adolescents with a history of migration in Germany: Results of the STRONGDATA-Kids project
Source: Bundesgesundheitsblatt Gesundheitsforschung Gesundheitsschutz. 2026 May 18;69(6):623–34. [Article in German] doi: 10.1007/s00103-026-04246-2 (PMC13212762; doi:10.1007/s00103-026-04246-2)
Supplement: Supplementary file 1 — Onlinematerial: Zusätzliche Abbildungen und Tabellen [file 103_2026_4246_MOESM1_ESM.pdf]

Onlinematerial: Zusätzliche Abbildungen und Tabellen

Tabelle A1: Parameter zur Beschreibung eingeschlossener Datenquellen

Allgemein	Population	Gesundheitsoutcome
• Name der Datenquelle	• Alter (bzw. Altersgruppen)	• Outcome 1
• Art: Routine/Survey	• Migrationsindikatorik<br>Geburtsland<br>Geburtsland (Eltern)<br>Staatsangehörigkeit<br>Staatsangehörigkeit (Eltern)<br>Aufenthaltsstatus<br>Einreisejahr<br>Deutschkenntnisse<br>Zuhause gesprochene Sprache	• Messinstrument
• Design: Längsschnitt/Querschnitt		• Outcome 2
• Primär-/Sekundärdatenquelle		• Messinstrument
• Datenhalter/Institution		• …
• Datenzugang		
• Datenumfang		
• Erhebungszeitraum		
• Periodizität		
• Geograf. Verteilung		

Abbildung A1: Scorecard zur Evaluierung von Gesundheitsdatensystemen

UNIVERSITÄT BIELEFELD
School of Public Health

ROBERT KOCH INSTITUT

Scorecard zur Evaluierung von Gesundheitsdatensystemen

Die folgende Scorecard wurde entwickelt, um:

- die Stärken und Schwächen der Datenquellen und -systeme zur psychischen Gesundheit von Kindern und Jugendlichen mit eigener oder elterlicher Migrationserfahrung in Deutschland zu charakterisieren,
- zentrale Dimensionen der Datenqualität, Relevanz, Zugänglichkeit und Nutzbarkeit für die Gesundheitsberichterstattung zu bewerten,
- Barrieren beim Zugang und der Nutzung dieser Datenquellen zu identifizieren,
- Verbesserungen vorzuschlagen, um die Zugänglichkeit, Qualität und Nutzbarkeit der Daten zu verbessern und an die Anforderungen der Gesundheitsberichterstattung anzupassen.

Zur Evaluierung der verschiedenen Dimensionen der Datenquellen wird eine fünfstufige Likert-Skala verwendet. Jedes Evaluierungskriterium wird am Ende des Dokuments erläutert. Die Scorecard ist flexibel und kann an andere Forschungsfragen angepasst werden.

Hinweise zur Nutzung:

1) Vorbereitung der Evaluierung

- Der Forschungsschwerpunkt oder das Forschungsziel wird festgelegt.
- Grundlegende Informationen zu den zu evaluierenden Datenquellen werden erfasst, einschließlich:
  - Name, Typ und verantwortliche Institution der Datenquelle
  - Zielpopulation, Datenumfang und geografische Abdeckung
- Optional können die Evaluierungskriterien und die Likert-Skala an die spezifischen Anforderungen des Forschungsbereichs angepasst werden (idealerweise mit Beispielen, die sowohl „stimme voll und ganz zu“ als auch „stimme überhaupt nicht zu“ veranschaulichen).

2) Durchführung der Bewertung

- Die Scorecard wird ausgefüllt, indem jede Dimension (z. B. Genauigkeit, Konformität) anhand der Likert-Skala bewertet wird (stimme überhaupt nicht zu / stimme nicht zu / Teils, teils / stimme zu / stimme voll und ganz zu; bei Bedarf können "nicht zutreffend" oder "unklar" verwendet werden).
- Zur besseren Kontextualisierung können Anmerkungen zu jeder Bewertung hinzugefügt werden.

3) Analyse der Ergebnisse

- Stärken, Schwächen sowie häufige Nutzungs- und Zugangsbarrieren jeder Datenquelle werden identifiziert.
- Bei Bedarf erfolgt ein Vergleich der Datenquellen.

1

Gefördert durch:
Bundesministerium für Gesundheit

Allgemeine Informationen

Name der Datenquelle*	
Inhalt und Zweck der Datenquelle	
Herausgeber/ verantwortliche Institution	
***Datenumfang/ Teilnehmende** *(gesamt – über alle Erhebungszeitpunkte hinweg)*	
Population*	
Geografische Abdeckung	
Erhebungszeitraum/ laufend*	
Anmerkungen:	

Fragen, die mit einem Stern (*) gekennzeichnet sind, sollten beantwortet werden.

Gefördert durch:
Bundesministerium für Gesundheit

UNIVERSITÄT BIELEFELD
School of Public Health

ROBERT KOCH INSTITUT

Scorecard Evaluierung

	Stimme überhaupt nicht zu	Stimme nicht zu	Teils / teils	Stimme zu	Stimme voll und ganz zu	Nicht zutreffend	Unklar
1. ***Genauigkeit***: *Die Datenquelle liefert genaue und präzise Daten.*<br>*Anmerkungen:*							
2. ***Konformität***: *Die Datenerhebung und -verarbeitung entspricht den relevanten Datenschutzgesetzen.*<br>*Anmerkungen:*							
3. ***Konsistenz***: *Die Daten sind über verschiedene Datensätze und Zeiträume hinweg konsistent.*<br>*Anmerkungen:*							
4. ***Zuverlässigkeit***: *Die Daten sind zuverlässig und gut dokumentiert.*<br>*Anmerkungen:*							
5. ***Aktualität***: *Die Daten sind ausreichend aktuell und zeitgemäß für die beabsichtigte Forschungsanalyse.*<br>*Anmerkungen:*							

Teilevaluierung – Zugang und Barrieren

6. *Die Daten können ohne* ***rechtliche***** Hindernisse abgerufen und genutzt werden.*<br>*Anmerkungen:*							
7. *Die Daten können ohne* ***finanzielle*** *Hindernisse abgerufen und genutzt werden.*<br>*Anmerkungen:*							
8. *Die Daten können ohne* ***strukturelle***** Hindernisse abgerufen und genutzt werden.*<br>*Anmerkungen:*							

* Rechtliche Barrieren beziehen sich auf gesetzliche Einschränkungen oder Rahmenbedingungen, die regeln, wie Daten genutzt oder geteilt werden dürfen (z. B. Datenschutzgesetze oder Vertraulichkeitsvereinbarungen), während strukturelle Barrieren technische oder organisatorische Hindernisse umfassen, die den Zugang zu den Daten erschweren (z. B. Authentifizierungsanforderungen).

Bundesministerium für Gesundheit

UNIVERSITÄT BIELEFELD
School of Public Health

ROBERT KOCH INSTITUT

Erklärung

Beispielhafte Fragen, die bei der Evaluierung der verschiedenen Dimensionen innerhalb der Bewertungsmatrix für Gesundheitsdatenquellen berücksichtigt werden könnten

Dimensionen	Indikatoren	Kernfragen
1. Genauigkeit	1.1. Methodologie (einschließlich Datenerhebung)<br>1.2. Detaillierungsgrad und regionale Auflösung<br>1.3. Ausrichtung an externen Benchmarks und Standards	*1.1.1. Wie wurden die Daten erhoben? Wurde eine robuste, standardisierte und wissenschaftlich gültige Methodologie verwendet, um Expositionen und Ergebnisse zu messen?*<br>*1.2.1. Wie detailliert sind die Daten? Wie hoch ist die Granularität und regionale Auflösung? Sind die Daten präzise genug für die beabsichtigte Analyse?*<br>*1.3.1. Stimmen die Daten mit externen Benchmarks oder Standards überein? Gibt es Unstimmigkeiten, wenn die Ergebnisse mit anerkannten Quellen oder etablierten Metriken verglichen werden?*
2. Konformität	2.1. Einhaltung der Datenschutzgesetze<br>2.2. Datenanonymisierung<br>3.3. Einhaltung ethischer Richtlinien	*2.1.1 Entspricht der Datenerhebungsprozess den relevanten Datenschutzgesetzen (z.B. GDPR/DSGVO)? Liegen explizite Genehmigungen und Zustimmungen vor?*<br>*2.2.1. Wurden die Daten ggf. anonymisiert? Wurden personenbezogene Daten korrekt behandelt?*<br>*2.3.1. Werden ethische Richtlinien bei der Datenerhebung und -nutzung beachtet? Wurden ethische Fragen oder Interessenkonflikte angesprochen?*
3. Konsistenz	3.1. Forschungsspezifische Indikatoren<br>3.2. Variabilität der Parameter<br>3.3. Standardisierung für Vergleichbarkeit	*3.1.1. Forschungsspezifisch: Welche spezifischen Indikatoren (z. B. Migrationsstatus) werden in den Daten verwendet? Sind sie klar definiert und konsequent über den gesamten Datensatz hinweg angewendet?*<br>*3.2.1. Werden Parameter konsistent über verschiedene Datensätze und über die Jahre hinweg erfasst, um die Vergleichbarkeit sowohl zwischen den Datensätzen als auch über die Zeit zu gewährleisten?*<br>*3.3.1. Gibt es standardisierte Definitionen oder Kodierungen für die erfassten Parameter, die die Vergleichbarkeit zwischen den Datensätzen erleichtern?*
4. Zuverlässigkeit	4.1. Transparenz und Dokumentation<br>4.2. Wiederholbarkeit der Datenerhebung	*4.1.1. Sind die Methoden zur Erhebung, Speicherung und Verarbeitung von Gesundheitsdaten gut dokumentiert?*<br>*4.2.1. Kann die Gesundheitsdatenquelle bei wiederholtem Abrufen oder Nutzung stets dieselbe Art von Daten liefern? Bietet dieselbe Gesundheitseinrichtung oder Datenbank bei Zugriff zu unterschiedlichen Zeiten zuverlässige und wiederholbare Ergebnisse?*
5. Aktualität	5.1. Aktualität der Daten<br>5.2. Häufigkeit der Aktualisierungen<br>5.3. Verzögerung von der Erhebung bis zur Veröffentlichung	*5.1.1. Wie aktuell sind die Daten im Verhältnis zur spezifischen Forschungsfrage?*<br>*5.2.1. Wie häufig werden die Daten aktualisiert?*<br>*5.3.1. Wie lange sind die Verzögerungen bei der Verfügbarkeit der Daten (von der Datenerhebung bis zur Veröffentlichung)?*

Gefördert durch:
Bundesministerium für Gesundheit

Dimensionen	Indikatoren	Kernfragen
6. Rechtliche Barrieren	6.1. Vertraulichkeitsvereinbarungen und Datenschutzgesetze<br><br>6.2. Regulierungs- und Rechtsrahmen, die die Datennutzung betreffen	*6.1.1. Gibt es Vereinbarungen oder Datenschutzgesetze, die die Nutzung oder Weitergabe der Daten einschränken?*<br>*6.2.1. Gibt es Vorschriften oder rechtliche Rahmenbedingungen, die beeinflussen könnten, wie die Daten genutzt werden dürfen, einschließlich Einschränkungen bei der Datenweitergabe zwischen Behörden oder Organisationen?*
7. Finanzielle Barrieren	7.1. Zugangskosten<br><br>7.2. Zusätzliche Kosten für die Datennutzung	*7.1.1. Gibt es Abonnementgebühren, Lizenzgebühren oder andere finanzielle Anforderungen für den Zugang zu den Daten?*<br>*7.2.1. Entstehen zusätzliche Kosten im Zusammenhang mit der Datennutzung, z. B. Kosten für Datenextraktion, Reisen zu Forschungseinrichtungen (wenn die Daten nur innerhalb interner Serverstrukturen zugänglich sind)?*
8. Strukturelle Barrieren	8.1. Strukturelle Zugangsbeschränkungen<br><br>8.2. Nutzer:innenunfreundlicher Zugang und Datenerkennung	*8.1.1. Gibt es spezifische strukturelle Zugangsbeschränkungen (z.B. Authentifizierungsanforderungen, geografische Einschränkungen), die den Zugang erschweren könnten?*<br>*8.2.1. Werden nutzer:innenunfreundliche Systeme oder Schnittstellen für den Zugriff auf die Daten bereitgestellt und/oder gibt es ineffektive Suchfunktionen oder unklare Kategorisierungen, die das Auffinden der Daten erschweren?*

Gefördert durch:
Bundesministerium für Gesundheit

UNIVERSITÄT BIELEFELD
School of Public Health

ROBERT KOCH INSTITUT

Indikatorik Migration und psychische Gesundheit

Hinweis: Es können die in der Studie verwendeten migrationsbezogenen Indikatoren markiert sowie die beobachteten psychischen Gesundheitsoutcomes und die entsprechenden Tools bzw. Erhebungsmethoden dokumentiert werden

Migrationsindikatorik		*(bitte ankreuzen)*
	Geburtsland	
	Geburtsland der Mutter	
	Geburtsland des Vaters	
	Staatsangehörigkeit(en)	
	Jahr der Einreise	
	Aufenthaltsstatus	
	Kenntnisse der deutschen Sprache	
	Zuhause gesprochene Sprache	
	Staatsangehörigkeit der Mutter	
	Staatsangehörigkeit des Vaters	
Psych. Gesundheit		*(bitte ausschreiben)*
	Outcome 1	
	Tool/Erhebungsmethode	
	Outcome 2	
	Tool/ Erhebungsmethode	
	Outcome 3	
	Tool/ Erhebungsmethode	
	Outcome 4	
	Tool/ Erhebungsmethode	

Für Fragen oder Feedback kontaktieren Sie bitte strongdatakids@rki.de

Gefördert durch:
Bundesministerium für Gesundheit

Abbildung A2: Häufigkeit der Kombination von Migrationsindikatoren in den Datenquellen (in %, n=37)

Kenntnisse der deutschen Sprache	Aufenthalts-status	Jahr der Einreise	Staats-angehörigkeit Eltern	Staats-angehörigkeit	Geburtsland Eltern	Geburtsland	Routine / Survey	Geburtsland	Geburtsland Eltern	Staats-angehörigkeit	Staats-angehörigkeit Eltern	Jahr der Einreise	Aufenthalts-status	Kenntnisse der deutschen Sprache
						94%	Geburtsland Eltern	84%						
					56%	56%	Staats-angehörigkeit	42%	42%					
				50%	78%	72%	Staats-angehörigkeit Eltern	74%	74%	32%				
			61%	44%	61%	61%	Jahr der Einreise	26%	26%	16%	26%			
		28%	28%	22%	28%	28%	Aufenthalts-status	0%	0%	5%	0%	0%		
	22%	28%	33%	22%	33%	28%	Kenntnisse der deutschen Sprache	21%	21%	5%	16%	11%	0%	
28%	28%	50%	61%	50%	78%	78%	zuhause gesprochene Sprache	47%	53%	37%	42%	16%	5%	11%

Tabelle A2: Ergebnisse der Scorecard-Evaluierung nach Dimensionen für alle eingeschlossenen Datenquellen (n=22)

Datenquelle	Genauigkeit	Konformität	Konsistenz	Zuverlässigkeit	Aktualität	Keine Barrieren		
						Rechtlich	Finanziell	Strukturell
Mikrozensus	5	5	4	5	5	5	3	4
Schuleingangsuntersuchung	3	4	3	5	3	3	0	3
PriCare	5	5	4	5	4	5	5	4
GKV-Abrechnungsdaten	3	5	4	4	4	3	3	3
Gesamtmedian	4.0	4.8	3.8	4.8	4.0	4.0	3.7	3.5
AID:A	5	5	5	5	4	5	5	5
AOK-Familienstudie 2022	5	0	5	4	4	0	0	0
BELLA	5	5	5	5	4	0	0	0
CILS4EU	4	5	4	5	5	5	4	4
COPSY	5	5	5	5	5	5	5	5
EU-SILC	5	5	4	5	4	5	5	4
GME	3	4	3	5	4	3	0	3
HBSC	5	4	4	5	4	3	5	3
IAB-BAMF-SOEP	4	5	4	5	4	5	5	5
IAB-BiB/FReDA-BAMF-SOEP	4	5	4	5	4	5	5	5
IAB-SOEP-Migrationsstichprobe	4	5	4	5	4	5	5	5
IDEFICS	4	4	5	4	4	5	0	0
Integrate ADHD	4	5	0	5	4	0	0	0
KIDA	4	5	0	5	4	2	4	3
KIGGS	5	5	5	5	4	4	5	5
NEPS	5	5	4	5	4	5	5	4
Pairfam	5	5	5	5	4	5	5	4
ReGes	4	5	5	4	4	5	5	5
Gesamtmedian	4.4	4.8	4.4	4.8	4.1	4.5	4.9	4.3
Interrater-Reliabilität (Spearman ρ)	0.59	0.55	0.48	0.53	0.51	0.80	0.84	0.75

** 1 = stimme überhaupt nicht zu; 2 = stimme nicht zu; 3 = teils/teils; 4 = stimme zu; 5 = stimme voll und ganz zu; 0 = nicht zutreffend/unklar*

*** Die Berechnung des Gesamtmedians schließt Werte Null aus*
